# Intriguing electronic structure and photocatalytic performance of blueP–SMSe and blueP–SeMS (M = Mo, W) van der Waals heterostructures

**DOI:** 10.1039/d0ra07000a

**Published:** 2020-10-15

**Authors:** S. Ahmad, Iftikhar Ahmad, N. Van, B. Amin

**Affiliations:** Department of Physics, University of Malakand Chakdara 18800 Pakistan; Gomal University 29220 DI Khan Pakistan; Institute of Research and Development, Duy Tan University Da Nang 550000 Vietnam; Department of Physics, Abbottabad University of Science & Technology Abbottabad 22010 Pakistan binukhn@gmail.com

## Abstract

Van der Waals (vdW) combination of two dimensional (2D) materials in the form of a heterostructure is a widely accepted tool for tailoring properties and designing novel nanoelectronic and energy harvesting applications. The stacking geometry and electronic and photocatalytic properties of vdW heterostructures based on blueP and Janus SMSe and SeMS (M = Mo, W) monolayers are investigated by using first principles calculations. Two alternate stacking configurations of both heterostructures with an alternative order of chalcogen atoms in SMSe and SeMS (M = Mo, W) are constructed and found to be energetically and thermally stable. The feasible stackings of both heterostructures exhibit type-II band alignment (except blueP–SeMoS), hence are promising for light detection devices. In particular, the suitable positions of the valence and conduction band edges of all the heterostructures (except blueP–SMoSe) are appropriate for standard redox potentials and are capable of splitting water into O_2_/H_2_O and H^+^/H_2_. The findings pave the way for potential applications of these heterobilayer systems in future nanoelectronics, optoelectronics and photocatalytic water dissociation.

## Abstract 2

The stacking geometry and electronic and photocatalytic properties of vdW heterostructures based on blueP and Janus SMSe and SeMS (M = Mo, W) monolayers are investigated using first principles calculations.

## Graphical abstract

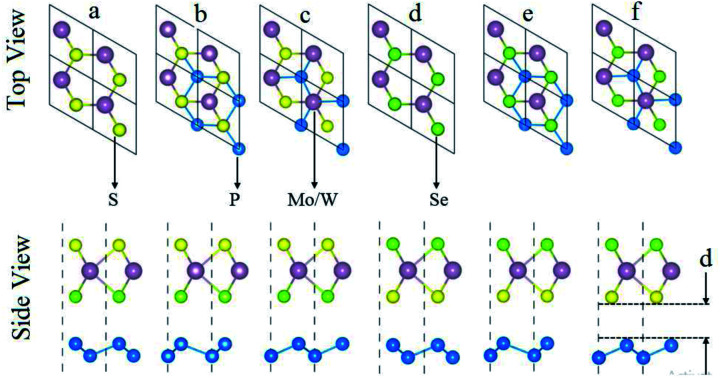

## 1 Introduction

The family of two-dimensional (2D) materials has been widely expanded to a variety of materials like transition metal dichalcogenides (TMDC),^1–4^ hexagonal boron nitrides (h-BN),^5,6^ phosphorene,^7,8^ and MXene.^9,10^ For instance, TMDC with the general formula MX_2_ (M = Mo, W; X = S, Se, Te) possess a semiconducting band gap nature, and have been extensively studied for versatile devices in optoelectronics, nanoelectronics and valleytronics.^11–13^ Very recently, Janus TMDC monolayers (with general formula MXY or XMY (M = Mo, W; X/Y = S, Se)) have been synthesized by breaking the structural symmetry of chalcogen atoms in MX_2_ through chemical vapor deposition (CVD) method.^14^ Janus XMY monolayers have an asymmetric structure, and exhibit a direct semiconducting band gap nature, giant Rashba spin splitting, exciting optoelectronic properties and photocatalysis of water.^15–18^

Blue phosphorus (blueP) and black phosphorus (blackP) with buckled honeycomb lattice symmetry have also been theoretically demonstrated as new 2D allotropes of phosphorous poly-types.^19–22^ Zhang *et al.*^23^ have successfully synthesized single layer blueP on an Au(111) substrate using molecular beam epitaxy technique. The graphene-like single layer buckled blueP has a flatter layer than puckered black phosphorus (blackP) and is predicted to exhibit a tunable band gap (2 eV) and electronic mobility (4.6 × 10^2^/4.7 × 10^1^ cm^2^ v^−1^ s^−1^ along armchair/zigzag directions at room temperature).^24,25^ Band gap engineering under external strain and electric field and the high charge capacities of single layer blueP have attracted a great deal of interest in nanoelectronics and capability for rechargeable Li-ion batteries.^26^

The combination of 2D materials *via* weak van der Waals (vdW) interactions to form heterostructures offers a way to study the controlled generation, combination or transportation of charge carriers for unique electronic, low power and ultrathin flexible photoelectronic devices.^27–29^ In vdW heterostructures, three different types of band alignments including type-I (symmetric), type-II (staggered), or type-III (broken) may be obtained for versatile device applications.^28–30^ However, heterostructures of type-II band alignment with conduction band minima (CBM) and valence band maxima (VBM) localized in two separate components lead to creation of interlayer optical excitations and reducing charge recombination rate in designing solar cells, novel optoelectronic and photovoltaic devices.^31^ A large variety of vdW heterostructures such as P/ZnO(SiC)^31^ and P/BSe^32^ with promising optoelectronic and photocatalytic water splitting properties have been theoretically and experimentally reported. Theoretical findings have revealed type-II band alignment with enhanced optoelectronic, spintronic and photocatalytic water splitting behavior in MoSSe/WSSe, GeC/Janus-TMDC and ZnO/Janus-TMDC heterostructures.^33–35^

In view of the above remarkable properties and acceptable lattice mismatch of blueP and Janus SMSe and SeMS (M = Mo, W) monolayers, the intriguing physical properties of the blueP–SMSe and blueP–SeMS (M = Mo, W) vdW stacking are investigated by first principles studies. In the present work, two different models of vdW heterostructures are formed with interchanging positions of the chalcogen atoms at the lower/top layers of the Janus monolayer. Each model has three possible stacking configurations. The feasible stacking configurations of the studied models of the heterostructures are found to be energetically and thermally stable. A detailed study of band structure, planar averaged charge density and photocatalytic behavior of the feasible stackings is carried out. Both heterostructures show type-II (except blueP–SeMoS) band alignment and are found most suitable for photocatalytic behavior. The findings reveal very promising applications of creating heterostructures for engineering optoelectronic and photovoltaic devices.

## 2 Computational details

Density functional theory (DFT) based calculations are performed using the projector plane wave (PAW) method^36^ as implemented in Vienna *ab initio* simulation package (VASP).^37,38^ DFT fails to include expansion caused by free energy of the system, hence structural comparison with systems at low temperature should make the minimum energy structure calculated by DFT+D (whatever dispersion correction) seem over-bound. Grimme DFT-D2 works best, providing the most binding out of the corrections. Therefore, vdW interactions are taken into account using the Grimme DFT-D2 method.^39^ The exchange-correlation functional proposed by Perdew–Burke–Ernzerhof (PBE)^40^ is used. In addition, the HSE06 (Heyd–Scuseria–Ernzerhof) functional is also adopted to obtain a better band gap value.^41^ The vacuum along the *z*-axis is set as 20 Å and the energy cutoff is chosen as 400 eV. The geometric relaxation is performed until the force and energy are converged to 0.001 eV Å^−1^ and 10^−4^ eV, respectively. A 6 × 6 × 1 *k*-point mesh is chosen for geometry relaxation and a 12 × 12 × 1 *k*-grid is further used for electronic structure calculations.

## 3 Results and discussion

After performing geometrical relaxation, the calculated lattice constant (bond length) of 2D blueP is 3.27 Å (2.25 Å for P–P), of SMSe is 3.26 Å (2.42 Å for S–M (M = Mo, W)), and of SWSe is 3.26 Å (2.53 Å for Se–M (M = Mo, W)) and are in good agreement with previous work.^14,24,29^ This indicates the accuracy of our computational approach. BlueP, SMSe and SeMS (M = Mo, W) layers have small lattice mismatches and identical hexagonal lattices thus attaining possible direction for experimental fabrication of blueP–SMSe and blueP–SeMS (M = Mo, W) vdW heterostructures in semiconductor applications.

**Fig. 1:**
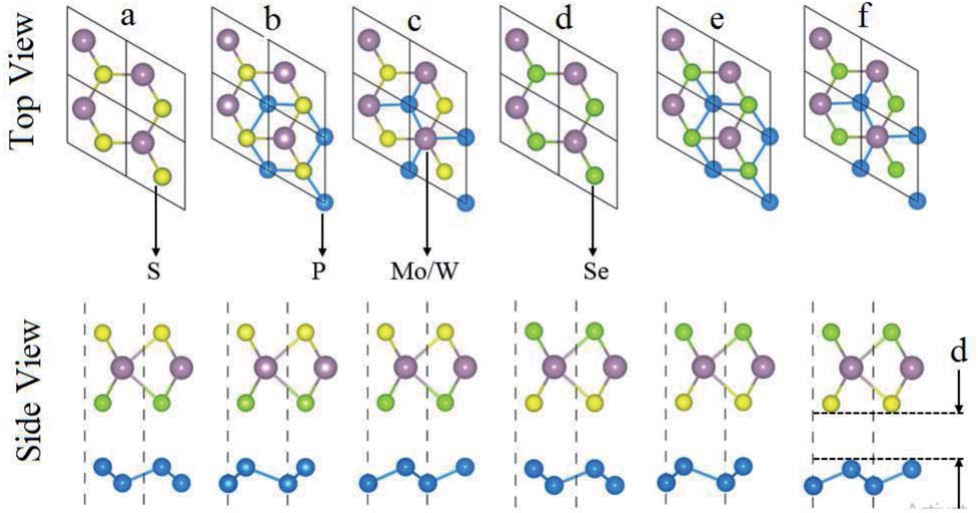
Top and side views of both models (P–SeMo(W)S and P–SMo(W)Se) with Se (a–c) and S (d–f) atoms located at the bottom surface of the Mo(W)SSe layer (details in text), and “*d*” represents the interlayer spacing.

Generally, the interfacial characteristics are sensitive to the local configuration and specified intercontacted atoms. Two different surfaces are possible for the construction of vdW heterostructures of blueP and single layer SMSe and SeMS (M = Mo, W). Therefore, two dissimilar models with interchanging positions of S and Se atoms on the opposite faces of Janus monolayer (SMSe and SeMS) were used forming three different possible stacking configurations, as shown in Fig. 1(a–c) and (d–f). In stacking a and stacking d, each blueP atom is set below the Mo, W atom and chalcogen atom. In stacking b and stacking e, one blueP atom is fixed below the chalcogen atom and a second blueP atom is placed at the hollow site. In stacking c and stacking f, an individual blueP atom is located below the Mo/W atom and another P atom occupies the hollow site.

**Fig. 2:**
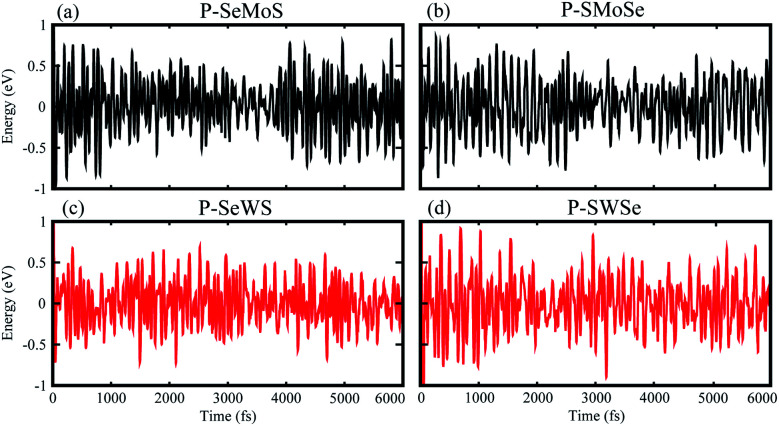
Thermal stability of P–SeMo(W)S and P–SMo(W)Se vdW heterostructures.

The binding energy of the heterobilayer system can be defined as *E*_b_ = *E*_blueP–SMSe,blueP–SeMS_ − *E*_blueP_ − *E*_SMSe,SeMS_, where *E*_blueP–SMSe,blueP–SeMS_ represents the total energy of the blueP–SMSe, blueP–SeMS heterostructure, *E*_blueP_ is the energy of the isolated single blueP layer and *E*_SMSe,SeMS_ represents the total energy of SMSe, SeMS monolayers. The theoretically predicted binding energy (*E*_b_), interlayer distance (*d*-spacing), optimized lattice constant (*a*), bond lengths (P–Mo, W and P–S, Se) of the studied heterostructures are presented in Table 1. The stacking patterns [configurations (b and e) of both models] exhibit very small interlayer spaces, which indicate highly energetic stability with very strong physical connection between blueP and MoSSe, WSSe. It is significant that the larger the covalent radius between S and Se, the larger the vdW attraction energy will be, which results in a very tiny change in the interlayer spacing. From Table 1, the smaller vertical distance “*d*-spacing” indicates that these heterobilayers contain vdW interactions. In addition, *ab initio* molecular dynamics (MD) calculations are performed to investigate the thermal stability of the studied heterobilayers. From Fig. 2, it is clear that no bond breaking or considerable variations in structure of these heterostructures is observed within 6 ps at room temperature. This indicates that all heterobilayers have stable geometry even at room temperature. Similar results are also observed in two dimensional graphene–gold interfaces.^42^

**Table 1:** Optimized lattice constant (*a*), binding energy (*E*_b_ in eV), interlayer distance (*d*_spacing_ in Å), bond length (P–P, Se–Mo/W and S–Mo/W), and band gap *E*_g_ (PBE and HSE06 functional) of P–Mo(W)SSe vdW heterostructures

Material	P–SeMoS	P–SMoSe	P–SeWS	P–SWSe
*a* (Å)	3.26	3.26	3.27	3.27
P–P (Å)	2.25	2.25	2.25	2.25
Se–Mo/W (Å)	2.53	2.53	2.53	2.53
S–Mo/W (Å)	2.42	2.42	2.24	2.24
*E*_b_/*d*_spacing_ (Å)	−0.325/3.15	−0.325/3.17	−0.348/3.25	−0.347/3.24
*E*_g_-PBE (eV)	1.02	0.93	0.92	0.90
*E*_g_-HSE06 (eV)	1.82	1.30	1.62	1.60
*E*_VB_ (eV)	1.74	1.58	1.60	1.59
*E*_CB_ (eV)	−0.084	0.076	−0.25	−0.013

**Fig. 3:**
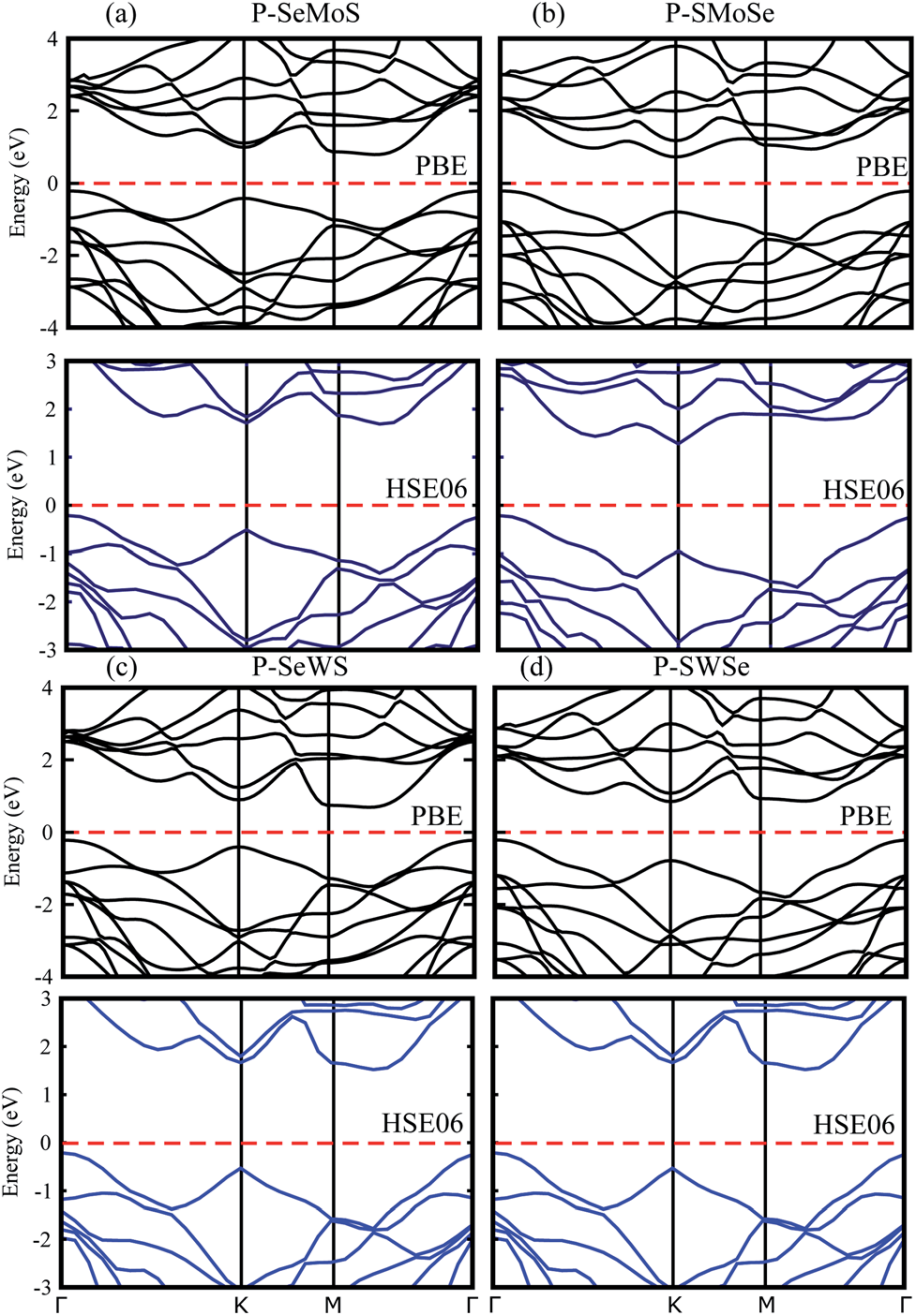
Electronic band structure of P–SeMo(W)S (a and c) and P–SMo(W)Se (b and d) with PBE (black) and HSE06 (blue) calculations.

Using both PBE and HSE06 functionals, blueP is found to be an indirect band gap semiconductor with the conduction band minimum (CBM) at the *Γ*–*K*-point and the valence band maximum (VBM) at the *M*–*Γ*-point of the Brillouin zone (BZ). The SMSe and SeMS are direct band gap semiconductors with both the CBM and VBM at the *K*-point of the BZ. The obtained PBE (HSE06) level band gap value for blueP is 2.19 (3.11) eV, for SMoSe is 1.47 (2.07) eV and for SWSe is 1.37 (2.07) eV. These results are in good agreement with previous literature.^14,24,29^ From Fig. 3, it is clear that blueP–SeMoS(blueP–SMoSe) and blueP–SeWS(blueP–SWSe) stackings possess indirect band gaps with the CBM lying between *M*–*Γ*(*K*) and the VBM at the *K*(*K*)-point of BZ. Band gap values of the reported vdW heterostructures are smaller than both monolayers, indicating that the formation of the vdW heterostructure reduces the band gap value. However, it is still larger than the minimum (1.23 eV) that is required for photocatalysis reactions, showing the potential application of the blueP/BSe heterostructure as a visible light photocatalyst.^43^

**Fig. 4:**
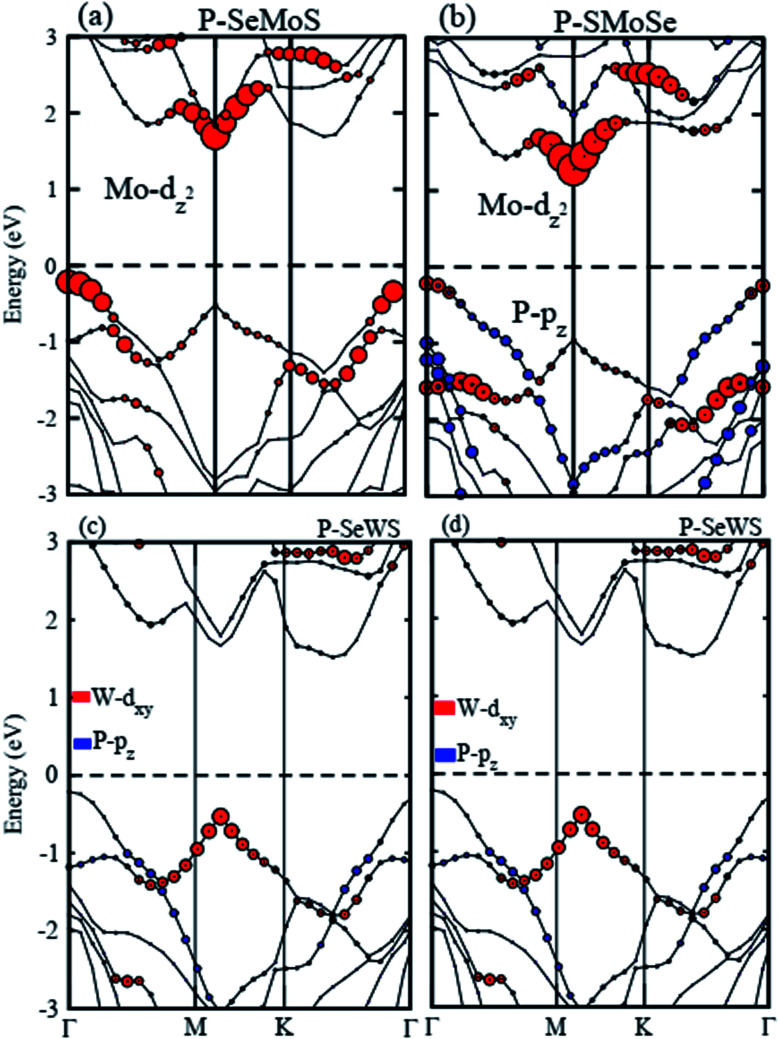
Weighted band structures of P–Mo(W)SSe vdW heterostructures.

Furthermore, careful observations have been made of the band alignments of blueP, SMSe, and SeMS vdW heterostructures by plotting the projected weighted bands, as shown in Fig. 4. Obviously, the P-p_*z*_ orbital mainly contributes in the VBM and the Mo-d_*z*^2^_ (W-d_*xy*_) orbital dominates the CBM in P–SMoSe and P–SeWS and P–SWSe. Certainly, the CBM and VBM in blueP–SMoSe, and blueP–SWSe are confined in blueP and SMoSe, SWSe monolayers, clearly showing type-II band alignment (known as staggered type). The VBM and CBM are localized from the Janus monolayers and blueP monolayer, respectively at the interface between these two layers. Such a type-II band-alignment spontaneously separates electrons and holes, enabling high efficiency optoelectronics and solar energy conversions.^43–46^ It has been discovered that type-II band alignments are mostly crucial in carrier separation of charges. This trend has been found in MoSSe–WSSe, GeC/Janus-TMDCs, ZnO/Janus-TMDCs, and SiC-TMDCs.^33–35^ Apparently, photogenerated electrons will transfer from blueP to SMoSe, SWSe and holes will move in the opposite direction from SMoSe, SWSe to blueP, this mechanism will minimize the rate of combination of charge. Hence, blueP–SMoSe, blueP–SWSe heterostructures must be thoroughly considered for applications in solar cells.^47^ However, the VBM and CBM are dominated by the Mo-d_*z*^2^_ orbital in P–SeMoS indicating type-I band alignment, which is desirable for laser or light emitting diodes.^48,49^

**Fig. 5:**
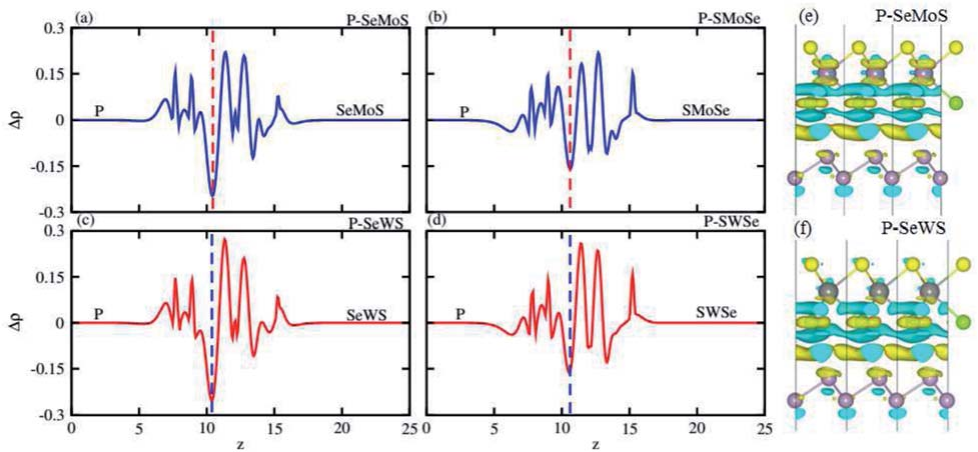
Planar-averaged charge density (a–d) and charge density difference (e and f) of PMo(W)SSe vdW heterostructures.

The charge density difference (Δ*ρ*) provides information about charge transfer across the interface and may be given as: Δ*ρ* = *ρ*_heterostructure_ − *ρ*_blueP_ − *ρ*_SMSe,SeMS_ where *ρ*_heterostructure_, *ρ*_blueP_, and *ρ*_SMSe,SeMS_ represent the charge density of the heterostructure, blueP and SMSe and SeMS monolayers, respectively. It is evident from Fig. 5(e and f) that charges are depleted from blueP and accumulated on SMoSe, SWSe (gain and loss of electrons is shown by cyan and yellow colors in Fig. 5), whereas the charge distribution is mainly present in the interfacial locality between blueP and nearby Se/S atoms, because of the difference between their electronegativities. To discuss charge redistribution in detail, the planar averaged charge density has been calculated along the *z*-direction perpendicular to the heterostructures as shown in Fig. 5(a–d). The values above zero (positive) in the figure indicate that the blueP gives electrons to SMSe, completing p-type doping in blueP and n-type doping in SMSe. Further, the Bader charge analysis technique^50^ is used for in depth concepts of the charge transfer mechanism from blueP to SMoSe, SWSe. It is noted that 0.32*e*(0.54*e*) and 0.21*e*(0.26*e*) fraction of charge has been transferred from blueP to SeMoS(SMoSe) and SeWS, SWSe monolayers. These indicated values show that in the calculated heterobilayer system, the blueP layer is an electron donor and SMoSe and SWSe layers are electron acceptors. This leads to a p(n)-type doping process in blueP(SMoSe and SWSe) in the studied heterobilayers. Furthermore, the transfer of charge between phosphorene and S/Se enhances an inner electric field partially detaches the photogenerated electrons in different atoms. This clearly shows a weak interaction between blueP and the SMSe monolayers; this characteristic has also been shown by GeC–Janus-TMDCs and ZnO–Janus-TMDCs.^34,35^

**Fig. 6:**
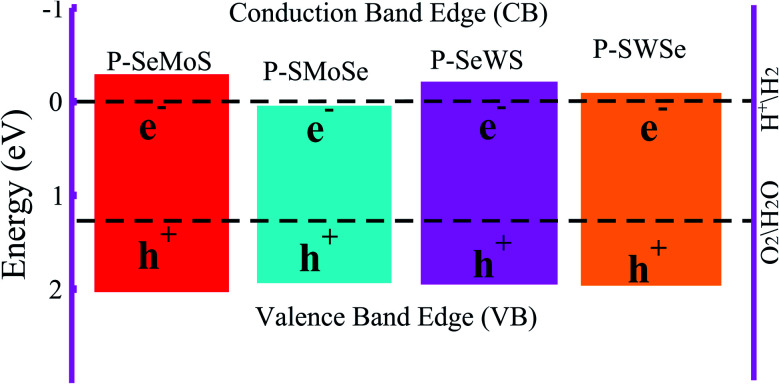
Valence and conduction band edges of both models of P–Mo(W)SSe vdW heterostructures with black dashed-lines representing the standard reduction (−4.44 eV) and oxidation (−5.67 eV) potentials for water dissociation into H^+^/H_2_ and O_2_/H_2_O, respectively.

The production of hydrogen gas under solar irradiation using a photocatalyst is in high demand for safe and environment friendly renewable energy sources. In general, the oxidation and reduction reaction for photocatalytic water splitting can be written as: 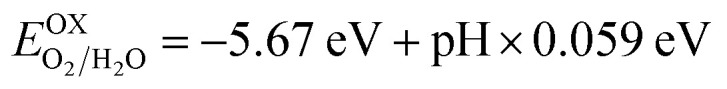 and 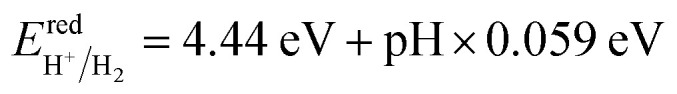.^51^ The photocatalysis of water splitting consists of transportation and separation of photogenerated charge carrier electrons from the material to H^+^/H_2_ and holes to oxygen molecules. It has been reported that materials with a band gap value greater than 1.23 eV are suitable for photocatalytic water splitting. These outcomes lead to the result that active O_2_ and H_2_ transformation are achievable under the influence of visible light, which leads to a reasonable photocatalytic mechanism.^52^ The positions of the band edges of the studied heterostructures as compared with the standard reduction (−4.44 eV) and oxidation (−5.67 eV) potentials for water splitting at pH = 0 are obtained using HSE06, see Fig. 6. It is evident from our previous work that both the VB and CB edges of blue-P, MoSSe and WSSe Janus monolayers can be seen to be more positive and more negative than the redox potential of O_2_/H_2_O and H^+^/H_2_ respectively, which is very near to the previous studies.^18,29,34,53^ This recommends the idea that water oxidation and reduction can be feasible thermodynamically for the possible heterostructures. Furthermore, the energy levels of the VB and CB for P–Mo(W)SSe heterobilayers are much larger than the general redox potential, which provides enough energy to drag the photogenerated charge carrier electrons and respective holes to dissociate water molecules into O_2_/H_2_O and H^+^/H_2_, this phenomenon rates this material as a good water splitting photocatalyst. A similar trend has also been demonstrated in GeC–Janus-TMDCs and ZnO–Janus-TMDCs heterobilayers.^34,35^ In contrast, P–SMoSe is not capable to perform the reduction reaction and is suitable for the oxidation reaction, as displayed in Fig. 6. Thus, we suggest that P–SMoSe, blueP–SWSe heterobilayers are exciting materials for water splitting and suitable for the commercial scale production of solar hydrogen.

## 4 Conclusion

In summary, we have calculated the structural, electronic and photocatalytic behaviors of vdW heterostructures of blueP and Janus SMSe and SeMS (M = Mo, W) using first principles calculations. Our results show that blueP heterostructures with SMoSe and SWSe through an alternate order of chalcogenides at opposite sides in SMSe and SeMS are energetically and thermally stable. All stackings (except P–SeMoS) show type-II band alignment which enables the charge carrier mechanism and are ideal for solar panels. Interestingly, the VB and CB edge positions of both models of the studied heterobilayer systems straddle the standard redox potentials and are useful for dissociation of water into O_2_/H_2_O and H^+^/H_2_. However, P–SMoSe is only suitable for performing the oxidation reaction. These theoretical findings provide a way for useful designing of optoelectronic and future renewable energy devices.

## Conflicts of interest

The authors declare that there are no conflicts of interest regarding the publication of this paper.

## Supplementary Material
